# Enhancing Diabetes Care in LMICs: Insights from a Multinational Consensus

**DOI:** 10.12669/pjms.39.7.8881

**Published:** 2023

**Authors:** Jose Carlos Miranda, S. Abbas Raza, Babatope Kolawole, Jahanzeb Kamal Khan, Atiba Alvi, Fariha Sadiq Ali, Ejim Emmanuel Chukwudi, Nanik Ram, Amira Oluwatoyin

**Affiliations:** 1 Dr. Jose Carlos Miranda President, CEO Southeast Asian Medical Center, Philippines. Email: jcendoc20@gmail.com; 2 Dr. S. Abbas Raza Shaukat Khanum Cancer Hospital & Research Center and National Hospital, Lahore, Pakistan. Email: sabbasraza@hotmail.com; 3 Dr. Babatope Kolawole Obafemi Awolowo University and Teaching Hospital (OAUTH) Ile-Ife, Nigeria. Email: bakolawole@gmail.com; 4 Dr. Jahanzeb Kamal Khan College of Physicians & Surgeons of Pakistan, Karachi, Pakistan. Email: jahanzebkk@yahoo.com; 5 Dr. Atiba Alvi Institute of Business Management, Karachi, Pakistan. Email: ateeba.shah@gmail.com; 6 Dr. Fariha Sadiq Ali Tabba Heart Institute, Karachi, Pakistan. Email: docham77@hotmail.com; 7 Dr. Ejim Emmanuel Chukwudi University of Nigeria Teaching Hospital, Ituku-Ozalla, Enugu State, Nigeria. Email: emmanuel.ejim@unn.edu.ng; 8 Dr. Nanik Ram The Aga Khan University Hospital, Karachi, Pakistan. Email: nanik.ram@aku.edu; 9 Dr. Amira Oluwatoyin Lagos University Teaching Hospital Idi-Araba, Nigeria. Email: toyinamira@yahoo.com

**Keywords:** Diabetes Care, LMICS, Consensus, Disease Management, International Collaboration

## Abstract

The International Cardio-Metabolic Forum held a plenary session to establish a multinational consensus on the challenges faced in diabetes management within lower-middle-income countries (LMICs) and their potential solutions. Stakeholders, including patients, family/caretakers, healthcare professionals, and healthcare policymakers & organizations, participated in discussions. The audience of 280 doctors from 15 different countries (Pakistan, Qatar, Sri Lanka, Kenya, Myanmar, Georgia, Nigeria, Philippines, Uzbekistan, Iraq, Tanzania, Cambodia, Kazakhstan, South Sudan and Libya) was divided into 4 groups led by Group Leaders to represent each stakeholder group. Questionnaires addressing key challenges and solutions specific to each group were used to facilitate consensus development. Participants voted on relevant options based on their clinical experience. SLIDO software was used for polling, generating separate results for each group. The insights shared by healthcare professionals highlighted the importance of improving medication accessibility and cost-effectiveness for patients, emphasizing the need for adherence to treatment plans and lifestyle modifications. The significance of balanced nutrition with low glycemic index food for enhancing quality of life was recognized. Caregivers of diabetic patients with comorbidities face increasing demands for care, particularly in relation to age-related milestones. Healthcare professionals emphasized the challenges posed by cultural beliefs and health awareness, underscoring the importance of teamwork and early referral for managing comorbidities. Healthcare policymakers need to focus on disease education, awareness programs, screening guidelines, and advocacy for community and clinical screening. By addressing these challenges, a more comprehensive and effective approach to diabetes management can be achieved in LMICs, ultimately improving outcomes for individuals with diabetes.

## INTRODUCTION

Non-communicable diseases (NCDs) such as diabetes, cardiovascular disease, cancer, and chronic respiratory illness are recognized worldwide as the leading causes of death and disability^1^. Diabetes, specifically, is ranked as the eighth leading cause of death and affects more than 8% of the global adult population^2^. Type-2 diabetes (T2D) is the most prevalent form of diabetes, characterized by inadequate insulin production resulting in high blood sugar levels^2^. Notably, T2D disproportionately impacts socioeconomically disadvantaged populations and immigrants, particularly in high-income countries (HICs), highlighting its association with socioeconomic status 2.

**Figure 1:**
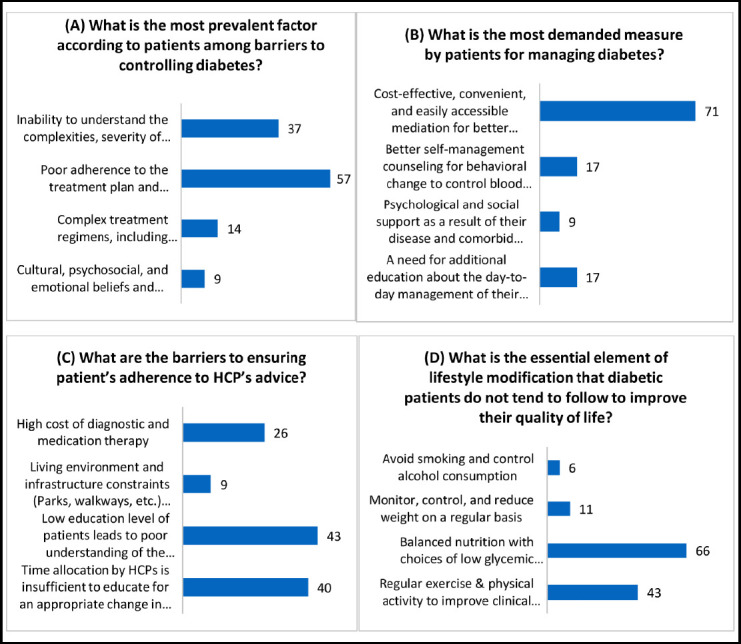
Responses obtained from the healthcare providers related to challenges faced by patients in for Diabetes management.

**Figure 2:**
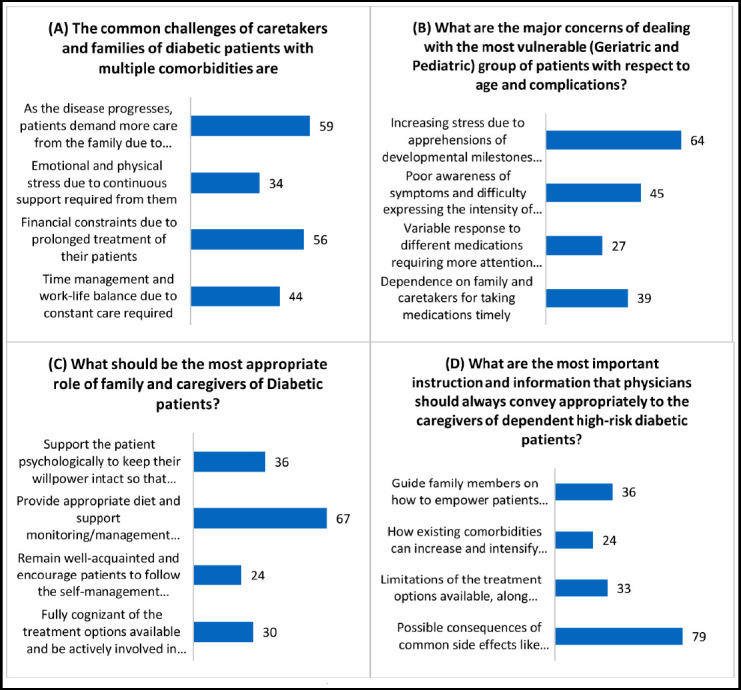
Responses obtained from the healthcare providers related to challenges faced by Family & Caretakers of the patients in Diabetes management.

**Figure 3:**
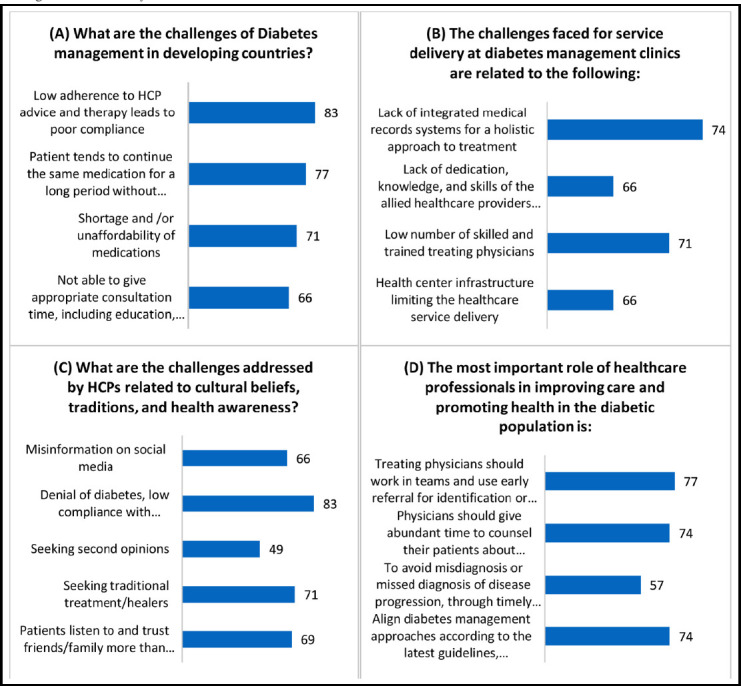
Responses obtained from the healthcare providers related to challenges faced by Healthcare Professionals for Diabetes management.

**Figure 4:**
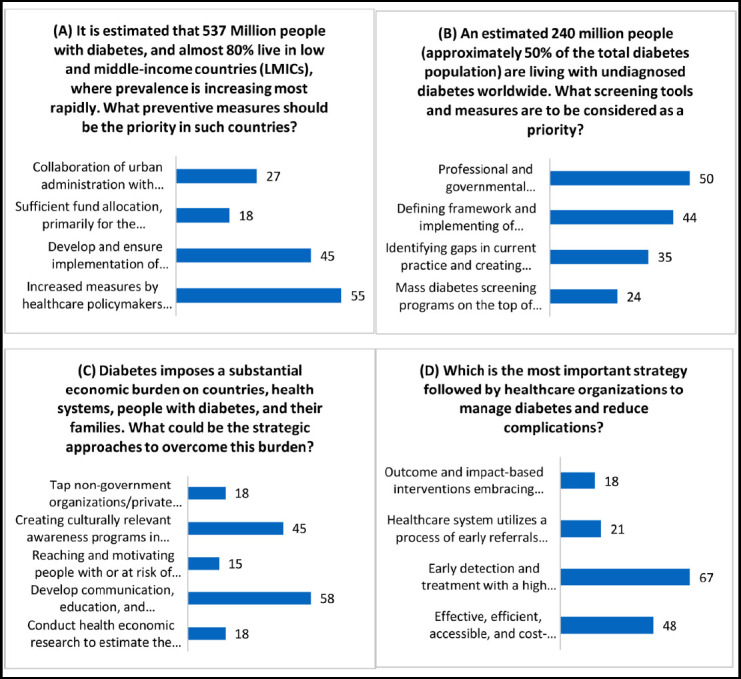
Responses obtained from the healthcare providers related to Healthcare Policymakers & Organizations for Diabetes management.

According to the World Health Organization, diabetes prevalence has been more pronounced in low- and middle-income countries (LMICs) compared to high-income countries^3^. Diabetes is associated with severe complications, including blindness, kidney failure, heart attacks, stroke, and lower limb amputation. Between 2000 and 2019, there was a 3% increase in age-specific mortality rates related to diabetes, and in 2019 alone, diabetes and diabetes-related kidney disease accounted for an estimated 2 million deaths^3^.

Patients with diabetes in low- and middle-income countries (LMICs) experience a number of obstacles that make it difficult for them to receive the best care possible, which leads to inadequate diabetes control and the emergence of disease-related comorbidities.^4^. The increased likelihood of a late diagnosis, which makes the situation even worse, is one of the main problems. Moreover, it might be challenging for people with diabetes to access and afford conventional and specialized healthcare services in many LMICs due to a shortage of qualified medical personnel and well-equipped clinics. As a result, less than 10% of patients in LMICs successfully manage their cholesterol, blood pressure, or glycemic control, with the majority of patients in these populations failing to do so^5^. Compared to high-income countries (HICs), LMICs experience a larger percentage of early fatalities brought on by high blood glucose levels. This emphasizes the critical need for efficient diabetes care models designed especially for LMICs, taking into account the multiple treatment barriers inherent in these under-resourced healthcare environments.^6^

Effective strategies exist for preventing or delaying the onset of Type-2 diabetes, focusing on lifestyle modifications such as adopting a healthy diet, engaging in regular physical activity, maintaining normal body weight, and avoiding tobacco use. Implementing these measures is crucial, as they have been proven to be effective in preventing or delaying the onset of T2D 7-10. Managing diabetes involves a combination of approaches, including dietary adjustments, regular physical activity, medication, and consistent screening and treatment of complications.

The Global Action Plan for the Prevention and Management of NCDs for the period 2013 to 2020 emphasizes the importance of prioritizing NCD prevention and addressing the social determinants underlying these diseases 11. To achieve these goals and ensure Universal Health Coverage (UHC), a people-centered primary healthcare approach that integrates community engagement is advocated^12^. Community engagement, as defined by the World Health Organization (WHO), involves establishing collaborative relationships among stakeholders to address health issues and promote well-being, ultimately leading to positive health outcomes 13.

In LMICs, strategies involving community engagement play a significant role in health promotion and disease prevention, including the management of NCDs. Community-clinical linkage models, such as the utilization of community health workers, have demonstrated effectiveness in delivering healthcare services to individuals with chronic diseases or those at risk, particularly in resource-limited settings^14^,^15^. However, community engagement in NCD prevention has not been extensively studied in LMICs.

Therefore, the objective of the proposed study was to gather insights from healthcare providers regarding challenges faced by each stakeholder involved in diabetes care in LMICs and establish a multinational consensus. The study aimed to identify the specific challenges experienced by patients, their families/caretakers, healthcare professionals, and healthcare policymakers/organizations. Through the utilization of questionnaires and a consensus-building process, the study seeks to develop a structured approach towards diabetes management that effectively addresses the key concerns raised by each stakeholder group.

## METHODOLOGY

A survey was carried out at the International Cardio-Metabolic Forum 2023 in Antalya, Turkey, to learn more about the difficulties faced by patients, their families and caregivers, medical professionals, and healthcare policymakers and organizations.

The audience consisted of about 280 doctors from 15 different nations including Pakistan, Qatar, Sri Lanka, Kenya, Myanmar, Georgia, Nigeria, Philippines, Uzbekistan, Iraq, Tanzania, Cambodia, Kazakhstan, South Sudan and Libya. The participants were then randomly divided into four groups to guarantee that each stakeholder group was represented. A Group Leader oversaw each group. By sending questionnaires created especially for each stakeholder group, consensus development was enhanced. These surveys had at least four questions, each of which addressed a known problem with illness management and potential remedies that would interest the designated stakeholder group.

Polling on the choices they felt were the most pertinent based on their clinical expertise was requested of all participants, including the group leaders. SLIDO software was used to carry out the polling procedure more efficiently. At the conclusion of the plenary session, this program allowed for the generation of distinct poll results, giving a thorough picture of the responses from each stakeholder group.

Each poll’s findings were combined, and the group leaders and other participants came to an agreement. This agreement served as the basis for creating a better-organized approach to diabetes care that addressed the major difficulties each stakeholder group experienced. This strategy aimed to improve diabetic patient outcomes and disease management tactics. In order to evaluate the survey data, SPSS Version 22.0 was used. The quantitative results were presented as frequencies and percentages, which made it easy to grasp the preferences and viewpoints of the participants.

## RESULTS

### Perceptions on Challenges Faced by Diabetes Patients

According to enrolled healthcare professionals (HCPs), one of the most prevalent factors hindering the effective management and motivation of individuals with diabetes, or those at risk, is poor adherence to the treatment plan and recommended lifestyle modifications (57%).

In terms of patient demands, the primary measure requested for managing diabetes is the availability of cost-effective, convenient, and easily accessible medications (71%).

One essential aspect of lifestyle modification that diabetic patients tend to neglect, despite acknowledging its significance in enhancing their quality of life, is balanced nutrition. Patients recognize the importance of making choices that involve consuming foods with a low glycemic index to avoid hypo- or hyperglycemia (66%).

### Perceptions on Challenges Faced by Caregivers of Patients

Caregivers and families of diabetic patients, particularly those with multiple comorbidities, commonly face the challenge of an increasing demand for care as the disease progresses (59%).

For vulnerable populations such as geriatric and pediatric patients, caregivers experience increasing stress due to concerns about developmental milestones in children and the worsening health conditions in the elderly (64%).

The most appropriate role identified for family members and caregivers of diabetic patients is to provide appropriate diet and support in monitoring and managing weight, thereby reducing the occurrence of hypo- and hyperglycemia events (67%).

Physicians should ensure they convey important instructions and information to caregivers of dependent high-risk diabetic patients in an appropriate manner. This includes educating them about the potential consequences of common side effects such as hypoglycemia, dehydration, and hypotension, as well as providing information about newer and safer treatment options available (79%).

### Perceptions on Challenges Faced by Healthcare Professionals

Healthcare professionals (HCPs) face several challenges in the management of diabetes, particularly in developing countries. One of the primary challenges identified is the low adherence of patients to HCP advice and therapy, resulting in poor compliance (83%).

In terms of service delivery at diabetes management clinics, a significant challenge reported by HCPs is the absence of integrated medical records systems that would allow for a more holistic approach to treatment (74%).

HCPs also encounter challenges related to cultural beliefs, traditions, and health awareness, particularly in relation to denial of diabetes and low compliance with medication and a healthy lifestyle (83%).

To enhance care and promote health among the diabetic population, it is emphasized that healthcare professionals should work in teams and prioritize early referrals for the identification or treatment of comorbidities, including cardiovascular and kidney diseases (77%).

### Perceptions on Challenges Faced by Healthcare Policymakers

Preventive measures in low- and middle-income countries (LMICs) should prioritize increased efforts by healthcare policymakers towards disease education and awareness programs, involving multiple stakeholders (55%).

According to healthcare providers’ perceptions, professional and governmental organizations play a crucial role in ensuring the development and application of screening guidelines, as well as advocating for screening in both community and clinical settings (50%).

To effectively address the burden of diabetes, healthcare policymakers and organizations should adopt a strategic approach that includes the development of communication, education, and engagement tools for coaches. These tools can be utilized to deliver the Diabetes Prevention Program, focusing on lifestyle and behavioral changes (58%).

Among the strategies employed by healthcare organizations to manage diabetes and reduce complications, healthcare providers prioritize early detection and treatment with a strong emphasis on achieving target HbA1C levels (67%).

## DISCUSSION

The present study aimed to gather insights from healthcare providers regarding the challenges faced by different stakeholders involved in diabetes care in low- and middle-income countries (LMICs) and establish a multinational consensus. The findings shed light on the perceptions of patients, caregivers, healthcare professionals, and healthcare policymakers, providing valuable information for developing a structured approach towards diabetes management in LMICs.

Our findings revealed several key challenges faced by diabetes patients in LMICs as per the perception of HCPs. Poor adherence to the treatment plan and recommended lifestyle modifications emerged as a prevalent barrier to controlling diabetes. This finding underscores the importance of addressing patient education and motivation to enhance compliance with therapy. Patients also expressed a strong demand for cost-effective, convenient, and easily accessible medications, which highlights the need for affordable and accessible healthcare services in LMICs. Additionally, the significance of balanced nutrition, including choices of low glycemic index food, was emphasized by patients to improve their quality of life and prevent hypo/hyperglycemia. Numerous studies have identified several variables leading to inadequate diabetic self-management. One key barrier, for instance, has been noted as the challenge of implementing and maintaining lifestyle modifications^16^.

Additionally, it has been discovered that ineffective communication interfaces within the healthcare system hinder successful self-management^17^. Limited availability of diabetes clinical supplies and compliance with adequate dietary guidelines have been connected to financial restrictions 18–20. Other research has honed in on particular facets of diabetic self-management and pinpointed associated obstacles. Researchers Nagelkerk et al. 21 and Ghimire^22^ discovered that people’s ability to engage in healthy eating and physical activity was hampered by their ignorance of specific diet plans and their perception that doing so is socially unacceptable. Further, depression symptoms and individual drug beliefs have been linked to lower adherence to diabetes treatments 23.

Diabetic patients’ caregivers in LMICs encounter a unique set of challenges. The study noted the rise in care needs as the illness worsens, especially in patients with several coexisting conditions. This research emphasizes the need to provide caregivers with proper support systems and resources because they are crucial in managing and assisting patients with complicated healthcare requirements. Concerns about children’s age-related developmental milestones and deteriorating health conditions in the elderly were also found, underscoring the significance of specialized care strategies for vulnerable populations. In a study, Zyberg and colleagues examined the challenges experienced by caregivers when caring for children with diabetes in various contexts. These difficulties span a range of areas, including regular blood glucose testing, developing and adhering to a nutrition plan, and monitoring the child’s physical activity 24. The obligation of caring for diabetic children at home frequently causes mental strain for the caregivers, according to a different study by Lindström et al. As parents work to offer the required care and support, managing the child’s diabetes can lead to emotional stress and exhaustion 25.

When it comes to managing diabetes, healthcare practitioners in LMICs have specific challenges. Significant barriers to efficient diabetes management have been identified as cultural attitudes, traditions, and low health knowledge. These elements play a part in the denial of diabetes, poor adherence to treatment and a healthy lifestyle, and false information spread on social media. In order to connect and educate patients in a meaningful way, the study emphasizes the significance of cultural sensitivity and knowledge among healthcare personnel. Additionally, in order to provide complete care and enhance patient outcomes, the importance of healthcare professionals working cooperatively in teams and using early referral for comorbidities, such as cardiovascular and kidney disorders, was underlined.

The difficulties faced by patients and healthcare professionals in managing diabetes mellitus (DM) in a low- and middle-income country (LMIC) were examined in a study by Nang et al. The findings brought to light a number of worries mentioned by medical staff regarding their lack of expertise and lack of confidence in managing diabetes. It was discovered that there were few diabetic care facilities available, and poor laboratory services presented additional difficulties^26^. Numerous low- and middle-income countries (LMICs) struggle with a lack of clinical staff and endocrinology specialists, a lack of adequate laboratory facilities, and restrictions on the accessibility to diabetes resources and treatments. Additionally, there is a lack of public understanding of diabetes and the health problems it is associated with, which has a negative impact on patient engagement in healthcare services and adherence to prescribed treatments. A significant number of patients are unable to purchase drugs and other essential medical supplies due to economic challenges 5,27.

Diabetes management presents difficulties for healthcare policymakers in LMICs. The report highlights the necessity for policymakers to put up more effort into illness education and awareness programs that involve a variety of stakeholders. Priority topics for healthcare policymakers include prevention measures, such as the implementation of screening recommendations and the promotion of screening in community and clinical settings. These results highlight the significance of a thorough and coordinated strategy involving decision-makers, healthcare providers, and community stakeholders to effectively address the burden of diabetes.

The development of scalable and sustainable methods for measuring diabetes risk, identifying those at high risk, and providing evidence-based interventions to this high-risk population are necessary for effective diabetes prevention at the population level 28. In order to address the social determinants of health, such as the standard of the food supply, built environment, and transportation networks, legislative reforms are also necessary 28,29. A significant impact can be had, and the risk of diabetes may be decreased for the entire community by systematically implementing policy reforms in these important areas. Furthermore, as they can support targeted diabetes preventive initiatives focused on high-risk populations, public health policies play an important role in boosting community health. These policies have the potential to increase the efficiency of research-based diabetes preventive measures by enhancing the state of health in communities. There is a need for coordinated efforts among clinical practices, health systems, and community organizations to optimize the impact of evidence-based preventative strategies. By facilitating the integration of diabetes screening and prevention services, this coordination would guarantee that those who are at risk receive the assistance and interventions they need.

The multinational consensus achieved through this study provides valuable insights for enhancing diabetes care in LMICs. The results underline how crucial it is for LMICs to have individualized interventions, complete healthcare systems, and greater illness education and awareness. The study also underlines the value of international cooperation and knowledge exchange in order to develop global standards for diabetes care.

### Limitations of the study:

Despite the valuable insights gained from this study, there are limitations to consider. The study focused on the perspectives of healthcare providers and did not directly involve patients and caregivers. Including the voices of patients and caregivers could provide a more comprehensive understanding of their experiences and needs. Furthermore, the study was carried out in the confines of a particular international forum, which can restrict the applicability of the findings in other contexts.

## CONCLUSION

The current study adds to the body of literature by shedding light on the challenges faced by various stakeholders involved in the management of diabetes in LMICs. The multinational consensus highlights the need for patient-centered approaches, integrated healthcare systems, and increased disease education and awareness. The findings provide a foundation for developing tailored strategies to address the specific challenges identified by patients, caregivers, healthcare professionals, and healthcare policymakers. By working collaboratively and implementing evidence-based interventions, it is possible to envisage a better future for diabetes care in LMICs and improve the overall health outcomes of individuals affected by the disease.
